# *Arcobacter butzleri* Induce Colonic, Extra-Intestinal and Systemic Inflammatory Responses in Gnotobiotic IL-10 Deficient Mice in a Strain-Dependent Manner

**DOI:** 10.1371/journal.pone.0139402

**Published:** 2015-09-25

**Authors:** Greta Gölz, Gül Karadas, Marie E. Alutis, André Fischer, Anja A. Kühl, Angele Breithaupt, Ulf B. Göbel, Thomas Alter, Stefan Bereswill, Markus M. Heimesaat

**Affiliations:** 1 Institute of Food Hygiene, Freie Universität Berlin, Berlin, Germany; 2 Department of Microbiology and Hygiene, Charité—University Medicine Berlin, Berlin, Germany; 3 Department of Medicine I for Gastroenterology, Infectious Disease and Rheumatology/Research Center ImmunoSciences (RCIS), Charité—University Medicine Berlin, Berlin, Germany; 4 Institute of Veterinary Pathology, Freie Universität Berlin, Berlin, Germany; Hungarian Academy of Sciences, HUNGARY

## Abstract

**Background:**

The immunopathological impact of human *Arcobacter* (*A*.) infections is under current debate. Episodes of gastroenteritis with abdominal pain and acute or prolonged watery diarrhea were reported for *A*. *butzleri* infected patients. Whereas adhesive, invasive and cytotoxic capacities have been described for *A*. *butzleri in vitro*, only limited information is available about the immunopathogenic potential and mechanisms of infection *in vivo*.

**Methodology/Principal Findings:**

Gnotobiotic IL-10^-/-^ mice were generated by broad-spectrum antibiotic treatment and perorally infected with the *A*. *butzleri* strains CCUG 30485 and C1 shown to be invasive in cell culture assays. Bacterial colonization capacities, clinical conditions, intestinal, extra-intestinal and systemic immune responses were monitored at day six and 16 postinfection (p.i.). Despite stable intestinal *A*. *butzleri* colonization at high loads, gnotobiotic IL-10^-/-^ mice were virtually unaffected and did not display any overt symptoms at either time point. Notably, *A*. *butzleri* infection induced apoptosis of colonic epithelial cells which was paralleled by increased abundance of proliferating cells. Furthermore *A*. *butzleri* infection caused a significant increase of distinct immune cell populations such as T and B cells, regulatory T cells, macrophages and monocytes in the colon which was accompanied by elevated colonic TNF, IFN-γ, nitric oxide (NO), IL-6, IL-12p70 and MCP-1 concentrations. Strikingly, *A*. *butzleri* induced extra-intestinal and systemic immune responses as indicated by higher NO concentrations in kidney and increased TNF, IFN-γ, IL-12p70 and IL-6 levels in serum samples of infected as compared to naive mice. Overall, inflammatory responses could be observed earlier in the course of infection by the CCUG 30485 as compared to the C1 strain.

**Conclusion/Significance:**

Peroral *A*. *butzleri* infection induced not only intestinal but also extra-intestinal and systemic immune responses in gnotobiotic IL-10^-/-^ mice in a strain-dependent manner. These findings point towards an immunopathogenic potential of *A*. *butzleri* in vertebrate hosts.

## Introduction

The motile and spiral-shaped gram-negative *Arcobacter (A*.*)* species belong to the family of *Campylobacteraceae* and can be isolated from a broad range of habitats. In animals *Arcobacter* spp. are mostly reported as gastrointestinal commensals [1]. Among the 19 so far described *Arcobacter* spp., *A*. *butzleri* and *A*. *cryaerophilus* have been rated as serious hazards for human health by the International Commission on Microbiological Specifications for Foods [2]. Given that detection of *Arcobacter* spp. may fail in applied bacteriologic routine diagnostic procedures, the prevalence of *Arcobacter* associated human diseases is not known so far. On the other hand, a huge number of single clinical cases and few outbreaks reported in the literature point towards an important role of these bacteria in causing intestinal diseases [3, 4]. Briefly, in retrospective studies several authors demonstrated that *Arcobacter* spp. are the fourth most common *Campylobacterales* species recovered from patients suffering from diarrhea [5–7]. Diseased patients have been shown to present symptoms of gastroenteritis including abdominal pain, acute diarrhea or prolonged watery diarrhea for up to two months [5, 6]. So far, limited information is available about the underlying mechanisms of infection and the host immune responses. Results from phenotypic assays revealed adhesive, invasive and cytotoxic capabilities of *A*. *butzleri* on several cell lines *in vitro* [8–14]. The barrier dysfunction caused by *A*. *butzleri* infection in monolayers of the human colon cell line HT-29/B6 highlights potential mechanisms by which diarrhea is induced in susceptible human hosts [15]. In order to study the pathogenic potential of *A*. *butzleri* in more detail *in vivo*, we monitored the colonization properties of two *A*. *butzleri* strains, for which invasive capacities have been shown in *in vitro* assays [8], and the subsequent host responses following peroral infection. We first infected conventional wildtype mice with distinct *A*. *butzleri* strains in order to investigate whether the strains are capable of colonizing the mice at all. We then included gnotobiotic IL-10^-/-^ mice in which the microbiota was virtually depleted by broad-spectrum antibiotic treatment into our experiments. In previous infection studies with enteric pathogens such as *Campylobacter (C*.*) jejuni* we could demonstrate that the physiological colonization resistance exerted by mice harboring a conventional microbiota prevented the animals from infection and could be overcome following eradication of the murine intestinal microbiota [16]. Furthermore, gnotobiotic IL-10^-/-^ mice developed infection-induced immunopathological key features of human campylobacteriosis within six days following peroral *C*. *jejuni* infection [17–20]. Given that *A*. *butzleri* (formerly termed *C*. *butzleri* [21]) is related to *C*. *jejuni*, we further unraveled colonization and immunopathological features of *A*. *butzleri* infection in the gnotobiotic IL-10^-/-^ mouse model. Corresponding results point towards an infectious and pro-inflammatory potential of *A*. *butzleri* which favours gnotobiotic murine models for the further study of pathogenicity factors of arcobacteriosis *in vivo*. By parallel investigation of two *A*. *butzleri* strains in the same experimental set-up also strain-dependent variances in the immunopathogenic potential could be observed.

## Methods

### Ethics statement

All animal experiments were conducted according to the European Guidelines for animal welfare (2010/63/EU) with approval of the commission for animal experiments headed by the “Landesamt für Gesundheit und Soziales” (LaGeSo, Berlin, registration number G0184/12). Animal welfare was monitored twice daily by assessment of clinical conditions.

### Mice

C57BL/6j wildtype and IL-10^-/-^ mice (in C57BL/6j background, B6) were bred and maintained in the facilities of the “Forschungseinrichtungen für Experimentelle Medizin” (FEM, Charité—Universitätsmedizin, Berlin, Germany) under specific pathogen-free (SPF) conditions. Gnotobiotic IL-10^-/-^ mice (with a virtually depleted gastrointestinal microbiota) were generated by broad-spectrum antibiotic treatment as described earlier [22]. In brief, mice were transferred to sterile cages and treated by adding ampicillin/sulbactam (1 g/L; Pfizer, Berlin, Germany), vancomycin (500 mg/L; Hexal, Holzkirchen, Germany), ciprofloxacin (200 mg/L; Hexal), imipenem (250 mg/L; Fresenius Kabi, Graz, Austria), and metronidazole (1 g/L; Braun, Melsungen, Germany) to the drinking water *ad libitum* starting at three weeks of age immediately after weaning and continued for 3–4 months before the infection experiment [19]. Three days before infection, the antibiotic cocktail was replaced by sterile tap water (*ad libitum*). Mice were continuously kept in a sterile environment (autoclaved food and tap water) and handeled under strict antiseptic conditions.

### *Arcobacter butzleri* infection of mice

Conventionally colonized wildtype and gnotobiotic IL-10^-/-^ mice (all female) were infected with approximately 10^9^ viable colony forming units (CFU) of either *A*. *butzleri* strain CCUG 30485 or strain C1 by gavage in a total volume of 0.3 mL PBS on two consecutive days (day 0 and day 1).

The *A*. *butzleri* reference strain CCUG 30485 was initially isolated from a fecal sample of a diarrheal patient [21], whereas the C1 strain was derived from fresh chicken meat [8]. Both *A*. *butzleri* strains were grown on Karmali-Agar (Oxoid, Wesel, Germany) for two days at 37°C under microaerobic conditions using CampyGen gas packs (Oxoid).

### Clinical Score

To assess clinical signs of *A*. *butzleri* infection on a daily basis, a standardized cumulative clinical score (maximum 12 points, addressing the occurrence of blood in feces (0: no blood; 2: microscopic detection of blood by the Guajac method using Haemoccult, Beckman Coulter / PCD, Krefeld, Germany; 4: overt blood visible), diarrhea (0: formed feces; 2: pasty feces; 4: liquid feces), and the clinical aspect (0: normal; 2: ruffled fur, less locomotion; 4: isolation, severely compromized locomotion, pre-final aspect)) was used [17].

### Sampling procedures

Mice were sacrificed by isofluran treatment (Abbott, Greifswald, Germany). Cardiac blood and tissue samples from colon, liver and kidney were removed under sterile conditions. Absolute large intestinal lengths were determined by measuring the distances from the ascending colon leaving the cecum to the rectum by a ruler. Intestinal samples from each mouse were collected in parallel for immunohistochemical, microbiological, and immunological analyses. Immunohistopathological changes were determined in colonic *ex vivo* biopsies immediately fixed in 5% formalin and embedded in paraffin. Sections (5 μm) were stained with hematoxylin and eosin (H&E) or respective antibodies for *in situ* immunohistochemistry as described earlier [19].

### Histopathological grading of intestinal lesions

To evaluate the severity of intestinal histopathological lesions, an established scoring scheme [23] with minor modifications was applied. In detail, the composition of immune cell infiltrates (0: none; 1: mononuclear cells; 2: mononuclear cell dominated, fewer neutrophils; 3: neutrophil dominated, fewer mononuclear cells), quantity of immune cell infiltrates (0: none; 1: mild; 2: moderate; 3: severe), vertical extent of inflammation (0: none; 1: mucosa; 2: mucosa and submucosa; 3: transmural), and horizontal extent of inflammation (0: no; 1: focal; 2: multifocal; 3: multifocal-coalescent; 4: diffuse) were assessed. Additionally the occurrence of crypt abscesses (0: no; 1: yes), epithelial hyperplasia (0: no; 1: yes), and erosions (0: no; 1: yes) was evaluated. The cumulative histologic scores ranged from 0 to 16 for colonic tissue.

### Immunohistochemistry

*In situ* immunohistochemical analysis of colonic paraffin sections was performed as described previously [16–18, 20]. Primary antibodies against cleaved caspase-3 (Asp175, Cell Signaling, Beverly, MA, USA, 1:200), Ki67 (TEC3, Dako, Denmark, 1:100), CD3 (#N1580, Dako, 1:10), F4/80 (# 14–4801, clone BM8, eBioscience, San Diego, CA, USA, 1:50), FOXP3 (FJK-16s, eBioscience, 1:100), and B220 (eBioscience, 1:200) were used. For each animal, the average number of positively stained cells within at least six high power fields (HPF, 0.287 mm^2^, 400 x magnification) were determined microscopically by a double-blinded investigator.

### Quantitative analysis of *Arcobacter butzleri* colonization and translocation

Before infection of gnotobiotic mice, absence of commensal intestinal microbiota was confirmed as described previously [16–20]. Viable *A*. *butzleri* were detected in feces or at time of necropsy (day 6 or 16 postinfection; p.i.) in luminal samples taken from the colon, dissolved in sterile phosphate buffered saline (PBS) and serial dilutions cultured on Karmali- and Columbia-Agar supplemented with 5% sheep blood (Oxoid) for two days at 37°C under microaerobic conditions using CampyGen gas packs (Oxoid). To quantify bacterial translocation, liver and kidney *ex vivo* biopsies were homogenized in 1 mL sterile PBS, whereas cardiac blood (≈200 μL) was directly streaked onto Karmali agar and cultivated accordingly. The respective weights of fecal or tissue samples were determined by the difference of the sample weights before and after asservation. The detection limit of viable pathogens was ≈100 CFU per g.

### Cytokine detection in serum and culture supernatants of intestinal and extra-intestinal *ex vivo* biopsies

Colonic *ex vivo* biopsies were cut longitudinally and washed in PBS. Kidney, liver or strips of approximately 1 cm^2^ colonic tissue were placed in 24-flat-bottom well culture plates (Nunc, Wiesbaden, Germany) containing 500 μL serum-free RPMI 1640 medium (Gibco, life technologies, Paisley, UK) supplemented with penicillin (100 U/ mL) and streptomycin (100 μg/ mL; PAA Laboratories). After 18 h at 37°C, culture supernatants and serum samples were tested for IFN-γ, TNF, MCP-1, IL-6, IL-12p70 by the Mouse Inflammation Cytometric Bead Assay (CBA; BD Biosciences, San Jose, CA, USA) on a BD FACSCanto II flow cytometer (BD Biosciences). Nitric oxide (NO) was determined by Griess reaction as described earlier [22].

### Statistical analysis

Medians and levels of significance were determined using Mann-Whitney test (GraphPad Prism v5, La Jolla, CA, USA) as indicated. Two-sided probability (*P*) values ≤ 0.05 were considered significant. Experiments were reproduced twice.

## Results

### Colonization capacities of *A*. *butzleri* in infected gnotobiotic IL-10^-/-^ mice

Given that mice can exhibit a strong colonization resistance against pathogens such as *C*. *jejuni* due to their host- and age-specific microbiota composition [16], we first addressed the question whether different *A*. *butzleri* strains (namely strain CCUG 30485 initially isolated from a diarrheal patient and strain C1 derived from fresh chicken meat) were able to stably establish intestinal colonization in wildtype mice with a conventional microbiota. Within 24 hours following the latest of two consecutive peroral infections with 10^9^ viable *A*. *butzleri* of either strain by gavage, mice expelled either pathogen from their intestines as indicated by culture-negative fecal samples and did not display any infection-induced clinical symptoms (data not shown).

**Fig 1:**
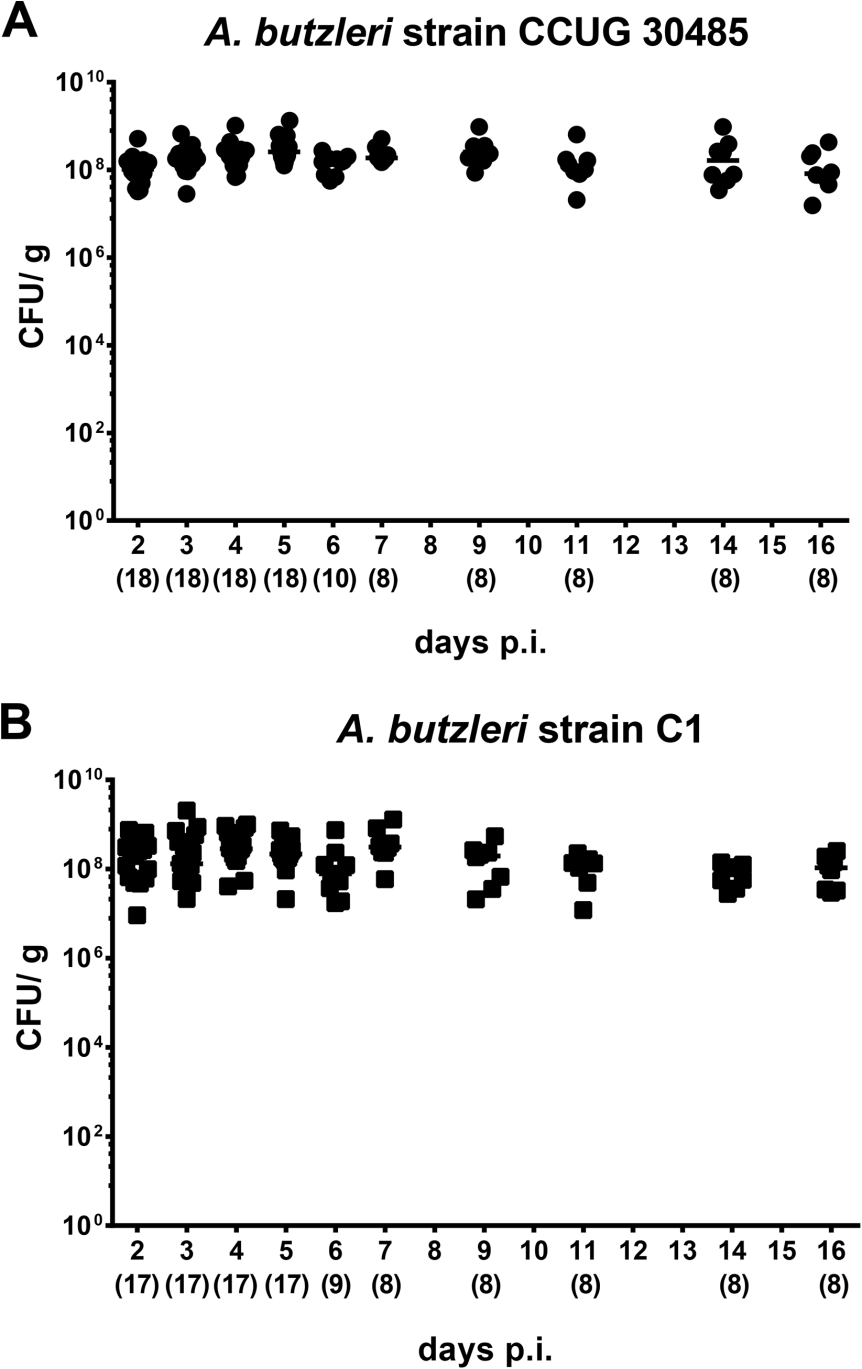
Fecal shedding of *A*. *butzleri* strains in orally infected gnotobiotic IL-10^-/-^ mice. Gnotobiotic IL-10^−/−^ mice were generated by antibiotic treatment and perorally colonized either with (**A**) *A*. *butzleri* strain CCUG 30485 or (**B**) strain C1. *A*. *butzleri* loads were determined in fecal samples (CFU, colony forming units per gram) over 16 days postinfection (p.i.) by culture. Numbers of analysed animals are given in parentheses. Medians (black bars) are indicated. Data were pooled from three independent experiments (S1 Table).

To overcome colonization resistance and investigate colonization as well as immunopathogenic properties of *A*. *butzleri in vivo*, we applied the gnotobiotic IL-10^-/-^ mouse model. This animal infection model was chosen since within one week upon *C*. *jejuni* infection gnotobiotic IL-10^-/-^ mice harbored high pathogenic loads and displayed non-selflimiting acute symptoms of infection-induced enterocolitis such as bloody diarrhea and wasting syndrome [17, 19]. Daily survey of *A*. *butzleri* loads in fecal samples revealed that following infection with 10^9^ viable *A*. *butzleri* strains CCUG 30485 or C1, gnotobiotic IL-10^-/-^ mice could be stably colonized with pathogenic loads of 10^8^ CFU per gram fecal sample (**Fig 1**). Despite high bacterial loads, however, mice did not display any overt infection-induced symptoms such as diarrhea or occurence of blood in feces at either time point (not shown).

### Induction of apoptosis in the colon of *A*. *butzleri* infected gnotobiotic IL-10^-/-^ mice

We next raised the question whether *A*. *butzleri* was able to induce intestinal inflammatory responses in colonized, but clinically unaffected mice. To address this, gnotobiotic IL-10^-/-^ mice were sacrificed as early as six days postinfection (p.i.) or to a later time point at day 16 p.i. Given that acute intestinal inflammation is accompanied by significant shortening of the intestinal tract [17, 22], we determined absolute lengths of the large intestines, but did not observe differences between mice infected with either strain, neither at day six nor at day 16 p.i., as compared to age- and sex-matched naive control animals (not shown).

**Fig 2:**
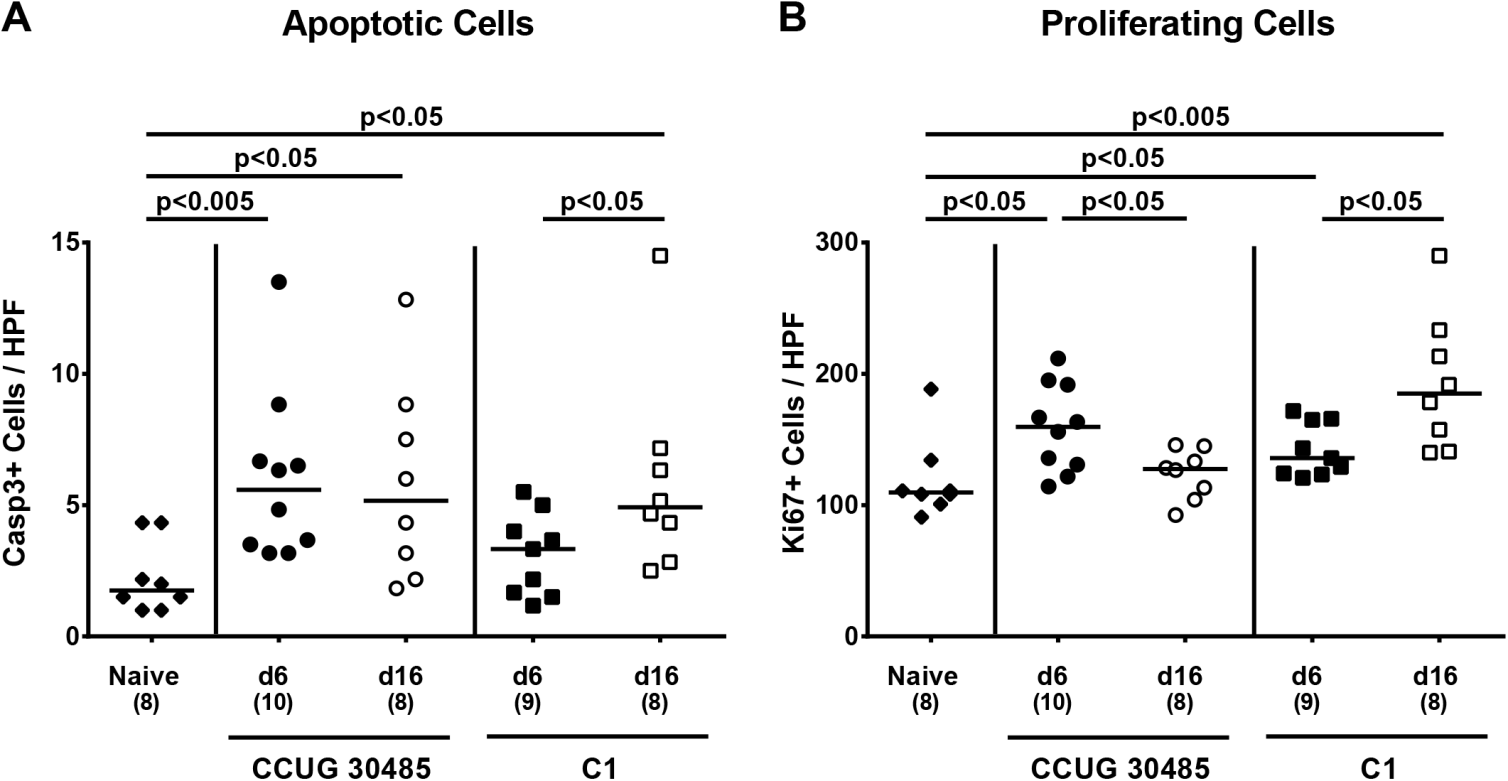
Apoptotic and proliferating cells in colon of gnotobiotic IL-10^-/-^ mice following *A*. *butzleri* colonization. Gnotobiotic IL-10^−/−^ mice were generated by antibiotic treatment and perorally infected either with *A*. *butzleri* strain CCUG 30485 (circles) or strain C1 (squares). Uninfected (naive) gnotobiotic IL-10^−/−^ mice served as negative controls (black diamonds). The average numbers of apoptotic (positive for caspase-3, Casp3, panel **A**) and proliferating cells (positive for Ki67, panel **B**) from at least six high power fields (HPF, 400 x magnification) per animal were determined microscopically in immunohistochemically stained colonic paraffin sections at day six p.i. (filled symbols) and day 16 p.i. (open symbols). Numbers of analyzed animals are given in parenthesis. Medians (black bars) and level of significance (p-value) determined by Mann-Whitney U test are indicated. Data were pooled from three independent experiments (S2 Table).

We further determined potential immunopathological responses in *A*. *butzleri* colonized animals. Histopathological changes in H&E stained colonic paraffin sections derived from with either *A*. *butzleri* strain colonized mice were rather subtle and did not differ at either time point as indicated by comparable histopathological scores (not shown). Since apoptosis is a commonly used diagnostic marker in the histopathological evaluation and grading of intestinal disease [16] and a hallmark of *C*. *jejuni* induced enterocolitis in gnotobiotic IL-10^-/-^ mice [17], we quantitatively assessed numbers of caspase-3+ cells within the colonic mucosa of colonized mice. At day six p.i. with the *A*. *butzleri* strain CCUG 30485, but not strain C1, mice displayed significantly higher numbers of apoptotic cells in the colonic epithelium as compared to uninfected controls (p<0.005; **Fig 2A**). At day 16 p.i., however, animals infected with either strain exhibited more than twofold higher caspase-3+ cell numbers in their colon versus naive mice (p<0.05; **Fig 2A**). As Ki67 comprizes a nuclear protein associated with and necessary for cellular proliferation [24], we stained colonic paraffin sections against Ki67 to determine proliferative measures of the colonic epithelium counteracting apoptosis following *A*. *butzleri* infection. Colonic epithelial Ki67+ cell numbers increased in strain CCUG 30485 infected mice until day six (p<0.05; **Fig 2B**), but reached levels observed in naive mice at day 16 p.i., whereas following C1 strain infection colonic proliferating cells further increased during the course of infection (p<0.05; **Fig 2B**).

### Large intestinal innate and adaptive immune cell responses in *A*. *butzleri* infected gnotobiotic IL-10^-/-^ mice

**Fig 3:**
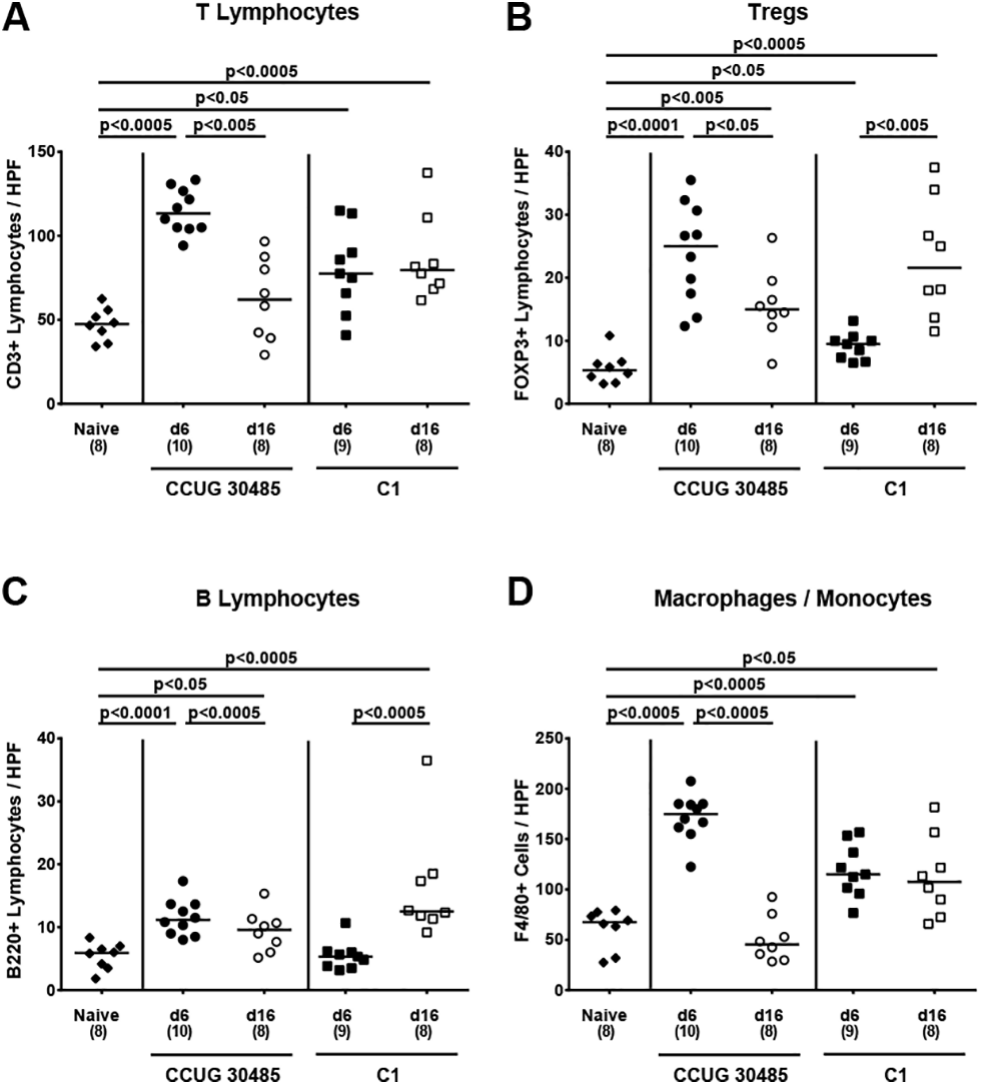
Colonic immune cell responses following *A*. *butzleri* infection of gnotobiotic IL-10^-/-^ mice. Gnotobiotic IL-10^−/−^ mice were generated by antibiotic treatment and perorally infected either with *A*. *butzleri* strain CCUG 30485 (circles) or strain C1 (squares). Uninfected gnotobiotic IL-10^−/−^ mice served as negative controls (black diamonds). The average number of cells positive for **(A)** CD3 (T lymphocytes), **(B)** FOXP3 (regulatory T cells, Tregs), **(C)** B220 (B Lymphocytes) and **(D)** F4/80 (macrophages and monocytes) from at least six high power fields (HPF, 400 x magnification) per animal were determined microscopically in immunohistochemically stained colonic paraffin sections derived from mice at day six p.i. (filled symbols) and day 16 p.i. (open symbols). Numbers of analyzed animals are given in parentheses. Medians (black bars) and significance levels as determined by the Mann-Whitney U test are indicated. Data were pooled from three independent experiments (S3 Table).

Since recruitment of pro-inflammatory immune cells to sites of inflammation is a well known key feature of enteric pathogen infection (e.g. campylobacteriosis [16]), we next quantitatively assessed the influx of innate and adaptive immune as well as of effector cells into the colonic mucosa and lamina propria by *in situ* immunohistochemical staining of large intestinal paraffin sections. Within six days following *A*. *butzleri* CCUG 30485 strain infection, CD3+ cell numbers (i.e. T lymphocytes) increased by more than twofold (p<0.0005; **Fig 3A**), but declined back to numbers observed in naive mice until day 16 p.i. In C1 strain infected mice, however, an increase in colonic T cells of approximately 25% in gnotobiotic IL-10^-/-^ mice could be observed until day six as well as day 16 p.i. (p<0.05 and p<0.0005, respectively; **Fig 3A**). Numbers of FOXP3+ regulatory T cells (Tregs) were higher in the large intestines of gnotobiotic IL-10^-/-^ at either time point and irrespective of the *A*. *butzleri* strain (p<0.05–0.0001; **Fig 3B**). Whereas during C1 strain infection Tregs further increased until day 16 p.i., lower FOXP3+ cell numbers could be detected in colons of CCUG 30485 strain infected mice at day 16 as compared to day six p.i. (p<0.05; **Fig 3B**). When compared to naive mice, numbers of B220+ B lymphocytes were significantly higher in CCUG 30485 strain infected mice, both at days six and 16 p.i. (p<0.0001 and p<0.05, respectively; **Fig 3C**), and 16 days following C1 strain infection (p<0.0005; **Fig 3C**). Interestingly, kinetic changes of F4/80+ macrophages and monocytes in CCUG 30485 and C1 strain infected mice were comparable to those seen with CD3+ cells: Whereas in colons of C1 strain infected mice F4/80+ cell numbers increased by approximately 50% until day 6 following infection and remained on comparable levels until day 16 p.i. (p<0.0005 and p<0.05, respectively; **Fig 3D**), numbers of colonic macrophages and monocytes in CCUG 30485 strain infected animals peaked at day six p.i. and reached naive levels ten days thereafter (**Fig 3D**).

### Induction of large intestinal pro-inflammatory immune responses in *A*. *butzleri* infected gnotobiotic IL-10^-/-^ mice

**Fig 4:**
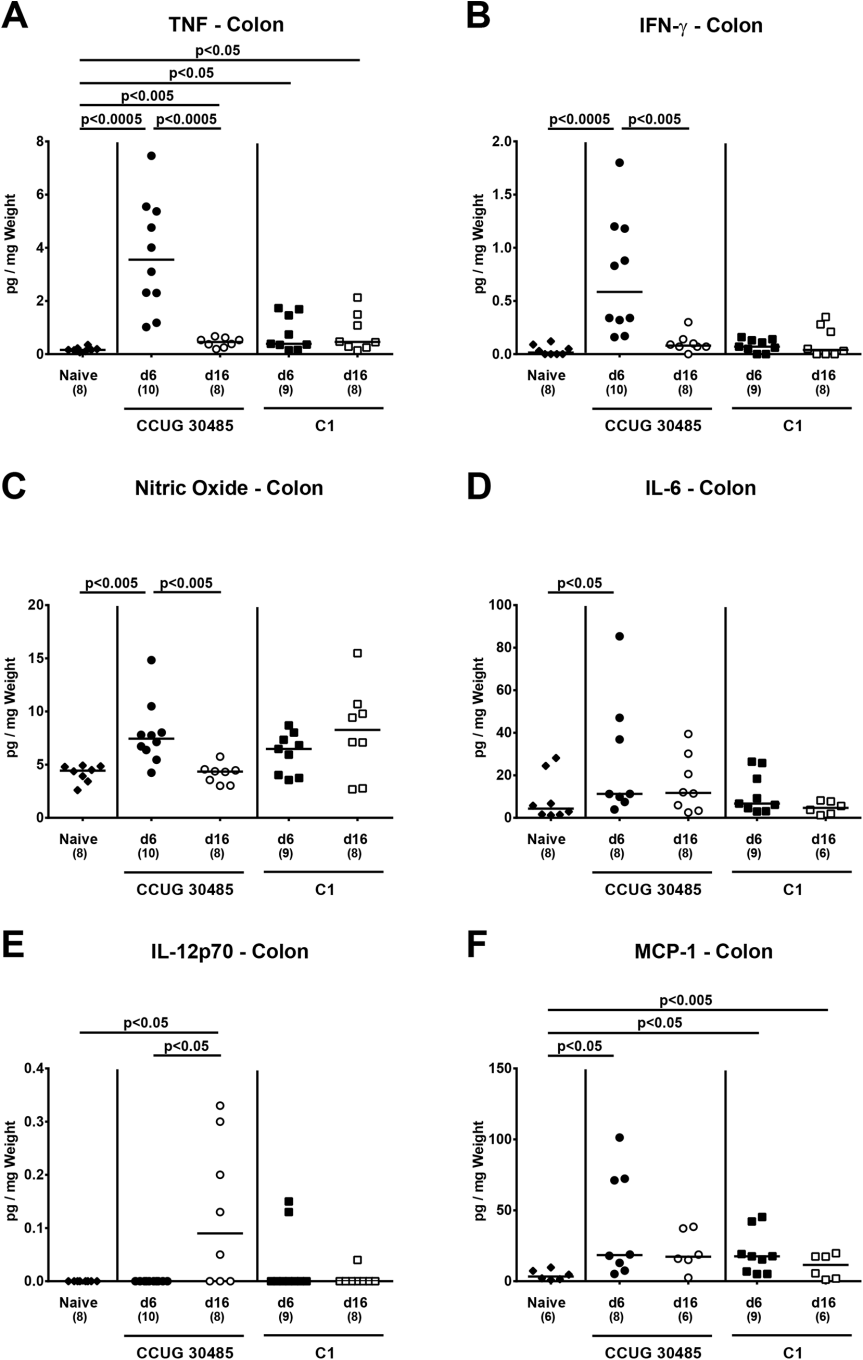
Colonic pro-inflammatory mediator responses following *A*. *butzleri* infection of gnotobiotic IL-10^-/-^ mice. Gnotobiotic IL-10^−/−^ mice were generated by antibiotic treatment and perorally infected either with *A*. *butzleri* strain CCUG 30485 (circles) or strain C1 (squares). Uninfected (naive) gnotobiotic IL-10^−/−^ mice served as negative controls (black diamonds). Concentrations of **(A)** TNF, **(B)** IFN-γ, **(C)** nitric oxide (NO), **(D)** IL-6, **(E)** IL-12p70 and **(F)** MCP-1 were determined in supernatans of *ex vivo* colonic biopsies at day six p.i. (filled symbols) and day 16 p.i. (open symbols). Numbers of analyzed animals are given in parentheses. Medians (black bars) and significance levels as determined by the Mann-Whitney U test are indicated. Data were pooled from three independent experiments (S4 Table).

In order to further assess large intestinal inflammatory sequelae of *A*. *butzeri* infection, we next measured pro-inflammatory cytokines in colonic *ex vivo* biospies. TNF protein levels increased upon *A*. *butzleri* infection with either strain and at both time points (p<0.05–0.0005; **Fig 4A**). Interestingly, TNF levels were more than 20 times higher in CCUG 30485 strain infected mice at day six p.i. as compared to naive mice, but declined significantly, still to an elevated level, thereafter (p<0.0005; **Fig 4A**). In colons of C1 strain infected mice TNF levels remained on comparable levels between day six and day 16 p.i. (**Fig 4A**). Furthermore, IFN-γ, nitric oxide (NO), and IL-6 levels measured in colonic *ex vivo* biopsies increased until day six following CCUG 30485 strain (p<0.05–0.0005; **Fig 4B–4D**), but not C1 strain infection, whereas at day 16 p.i. respective mediators were comparable to those observed in colons of uninfected mice (**Fig 4B–4D**). At day 16, but not day six p.i., increased colonic IL-12p70 levels could be observed following CCUG 30485 strain, but not C1 strain infection (p<0.05; **Fig 4E**). Moreover, colonic MCP-1 levels increased following *A*. *butzleri* infection (p<0.05; **Fig 4F**) except for day 16 following CCUG 30485 strain infection.

Taken together, despite absence of overt clinical symptom, *A*. *butzleri* infected mice exhibited colonic apoptosis accompanied by increased abundance of proliferating cells compensating for the intestinal damage. Furthermore an increase in immune cell populations in the large intestines could be observed which was accompanied by increased local pro-inflammatory mediator expression upon *A*. *butzleri* infection.

### Lack of obvious bacterial translocation, but induction of extra-intestinal and systemic pro-inflammatory immune responses in *A*. *butzleri* infected gnotobiotic IL-10^-/-^ mice

**Fig 5:**
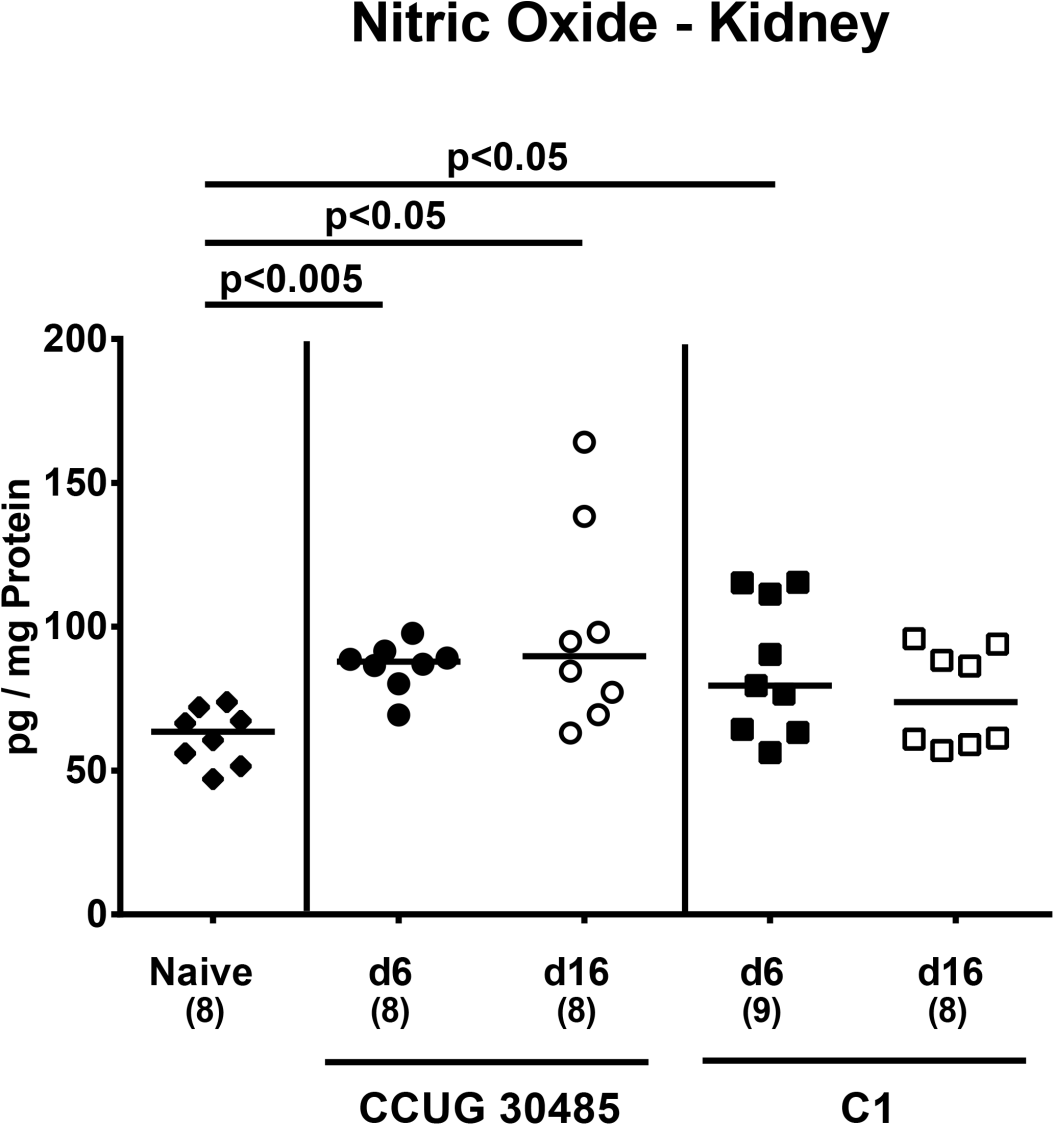
Nitric oxide secretion in renal *ex vivo* biopsies of *A*. *butzleri* infected gnotobiotic IL-10^-/-^ mice. Gnotobiotic IL-10^−/−^ mice were generated by antibiotic treatment and orally infected either with *A*. *butzleri* strain CCUG 30485 (circles) or strain C1 (squares). Uninfected (naive) gnotobiotic IL-10^−/−^ mice served as negative controls (black diamonds). Concentrations of nitric oxide (NO) were determined in supernatans of *ex vivo* kidney biopsies at day six p.i. (filled symbols) and day 16 p.i. (open symbols). Numbers of analyzed animals are given in parentheses. Medians (black bars) and significance levels as determined by the Mann-Whitney U test are indicated. Data were pooled from three independent experiments (S5 Table).

**Fig 6:**
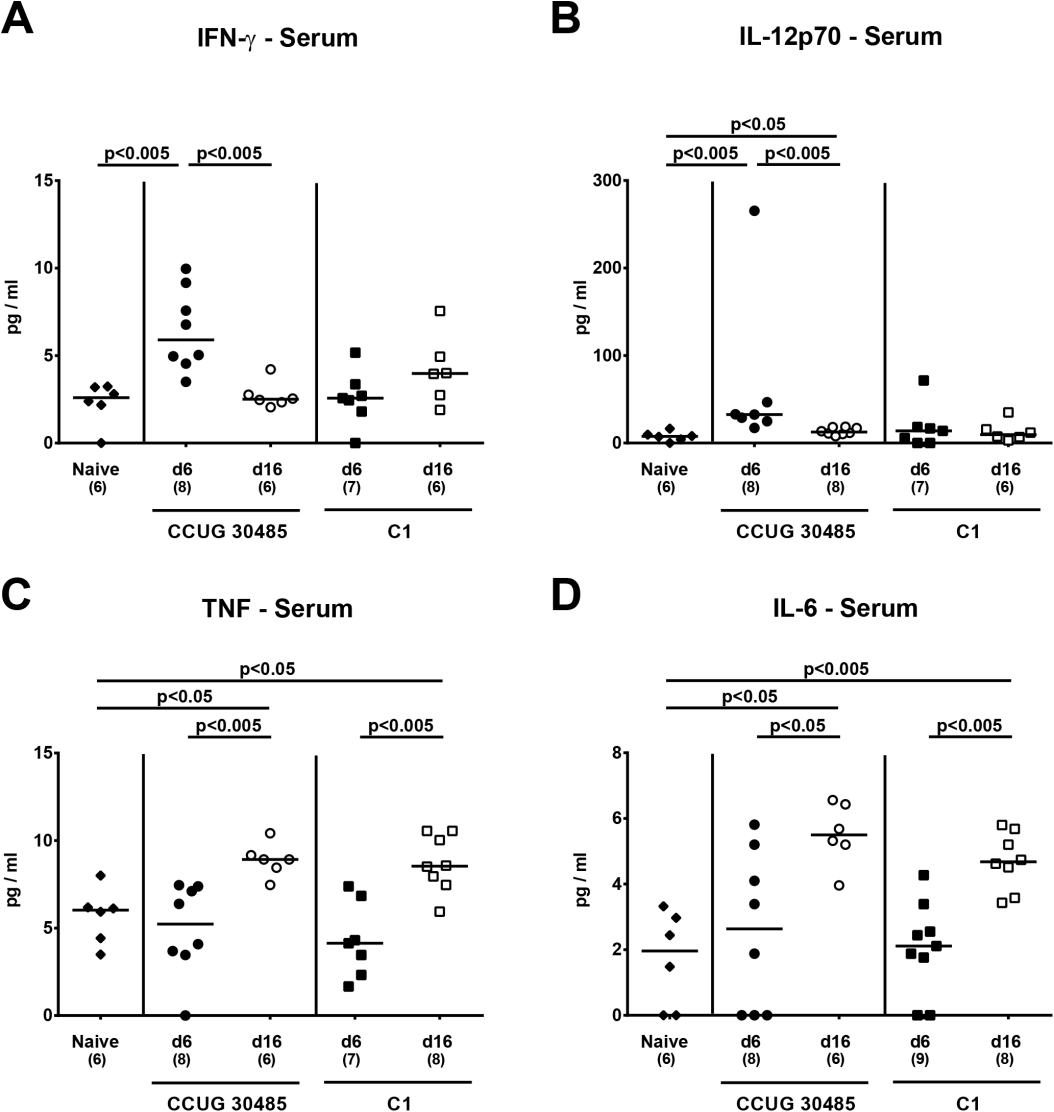
Systemic pro-inflammatory cytokine responses in *A*. *butzleri* infected gnotobiotic IL-10^-/-^ mice. Gnotobiotic IL-10^−/−^ mice were generated by antibiotic treatment and orally infected either with *A*. *butzleri* strain CCUG 30485 (circles) or strain C1 (squares). Uninfected gnotobiotic IL-10^−/−^ mice served as negative controls (black diamonds). Concentrations of **(A)** IFN-γ, **(B)** IL-12p70, **(C)** TNF and **(D)** IL-6 were determined in serum samples at day 6 p.i. (filled symbols) and day 16 p.i. (open symbols). Numbers of analyzed animals are given in parentheses. Medians (black bars) and significance levels as determined by the Mann-Whitney U test are indicated. Data were pooled from three independent experiments (S6 Table).

We next determined whether following *A*. *butzleri* infection bacterial strains might be able to translocate from the intestinal tract to extra-intestinal and systemic compartments. Irrespective of the *A*. *butzleri* strain, we were not able to detect any viable bacteria by direct plating (with a lower detection limit of 10^2^ CFU/g sample) from liver, kidney or cardiac blood–neither at day six nor at day 16 p.i. (not shown). In the following, we raised the question whether intestinal *A*. *butzleri* infection might induce pro-inflammatory immune responses at distant sites such as liver, kidney and serum. In H&E stained paraffin sections derived from liver and kidney samples no histopathological changes could be observed. Moreover, pro-inflammatory cytokine levels in livers of infected and naive mice did not differ at either time point (not shown). In kidneys, however, NO levels increased until day six following *A*. *butzleri* strain CCUG 30485 and C1 infection (p<0.005 and p<0.05, respectively; **Fig 5**), whereas at day 16 p.i. NO secretion in renal *ex vivo* biopsies were higher following CCUG 30485 (p<0.05; **Fig 5**), but not C1 strain infection. Remarkably, further distinct systemic pro-inflammatory cytokine responses could be observed in serum samples derived from *A*. *butzleri* infected gnotobiotic IL-10^-/-^ mice. As in colon, IFN-γ levels in serum increased multifold within six days following CCUG 30485 strain infection only, but decreased to naive levels until day 16 p.i. (p<0.005; **Fig 6A**). Furthermore, IL-12p70 serum levels increased following CCUG 30485, but not C1 strain infection peaking at day 6 p.i. (p<0.005; **Fig 6B**). Interestingly, serum levels of TNF and IL-6 increased rather late following CCUG 30485 and C1 strain infection as indicated by increased levels at day 16, but not day six p.i. (p<0.05–0.005; **Fig 6C and 6D**).

In summary, peroral *Arcobacter* infections resulted not only in local (i.e. intestinal), but also in significant extra-intestinal and systemic immune responses in a strain dependent manner.

## Discussion

The immunopathological impact of *Arcobacter* infections *in vivo* is under current debate. We here investigated the colonization and pro-inflammatory potential of peroral *Arcobacter butzleri* infection with two different strains in mice. As for other intestinal pathogens, mice harboring a conventional microbiota were protected from infection given that mice had expelled the respective *A*. *butzleri* strain within 24 hours p.i. despite dual peroral infection with high loads. This result is well in line with our previous *C*. *jejuni* infection studies showing that mice harboring a conventional microbiota were protected from colonization and expelled the pathogen within 48 hours p.i. [16]. To overcome physiological colonization resistance we applied gnotobiotic mice with a virtually depleted microbiota following broad-spectrum antibiotic treatment and chose the gnotobiotic IL-10^-/-^ mouse model that was recently shown very well suitable to elucidate immunopathological mechanisms of *C*. *jejuni* infection mimicking key featues of human campylobacteriosis such as acute enterocolitis within six days p.i. [17, 19, 20]. Interestingly, despite high intestinal *A*. *butzleri* loads, however, gnotobiotic IL-10^-/-^ did not display any overt symptoms such as diarrhea or occurence of blood in feces. Despite lack of clinical symptoms, *A*. *butzleri* induced colonic apoptosis which was paralleled by increased abundance of proliferating cells. This is well in line with an *in vitro* study showing a threefold increased epithelial caspase-3 dependent apoptosis induction in *A*. *butzleri* infected HT-29/B6 cells contributing to epithelial barrier dysfunction [15]. In another *in vitro* study, *A*. *butzleri* infection of THP-derived macrophages resulted in an increased activity of caspase-3 among caspase-7 and -8 which was accompanied by an upregulated expression of pro-inflammatory cytokines such as TNF, IL-6 and IL-12 [25] as shown in colon and serum of infected mice in our present *in vivo* study. Interestingly, despite initially increased caspase-3 levels, DNA damage was virtually absent suggesting potential counter-regulatory measures on the cellular level in THP-1 cells [25]. In line with this, we here demonstrate that *A*. *butzleri* induced large intestinal apoptosis was accompanied by increased numbers of Ki67+ proliferating cells in the colonic epithelium potentially counteracting the epithelial damage in the clinically unaffected infected mice. In previous *in vivo* studies, invasive capacities and virulence of *A*. *butzleri* were highly dependent on species and breed of the host and on the respective pathogenic strain [26]. In neonatal piglets, for instance, *A*. *butzleri* could be detected in small intestinal *ex vivo* biopsies and displayed rather invasive properties given that viable pathogens could be isloated from extra-intestinal organs such as liver, kidney, and even the brain after enrichment [27]. Eventhough we were unable to detect *A*. *butzleri* by direct plating from extra-intestinal compartments including liver and kidney in our study, we did detect higher NO levels in *ex vivo* biopsies derived from kidneys of infected mice underlining the potency of *A*. *butzleri* to induce not only intestinal but also extra-intestinal inflammatory collateral damages upon infection. Five days following oral infection with *A*. *butzleri*, albino rats were shown to present with diarrhea that was resolving within 21 days p.i., and small intestinal as well as hepatic necrosis [28]. The authors also described leukocytic infiltrates in the intestinal lamina propria, which is well in line with our results given that not only increased numbers of T and B cells, but also of regulatory T cells, macrophages and monocytes could be detected in the large intestinal mucosa and lamina propria of infected gnotobiotic IL-10^-/-^ mice. In another study by Adejisi et al., adult rats presented with watery diarrhea and electrolyte imbalances as well as increased concentrations of leukocytes and neutrophils in serum samples following infection with an *A*. *butzleri* strain in a pathogen-load dependent manner [29]. A systemic pro-inflammatory response following *A*. *butzleri* infection was also evident in our study given that in serum samples of infected mice a plethora of pro-inflammatory cytokines such as TNF, IFN-γ, IL-6 and IL-12 were upregulated. Diarrhea in *A*. *butzleri* shedding rats was self-limiting and could be observed for up to five weeks p.i. pointing towards a potential etiologic role in human diarrhea [30]. In contrast, *A*. *butzleri* was uncapable of colonizing conventional chicken and turkey poults, whereas Beltsville white turkeys displayed highly variable, and *A*. *butzleri* strain dependent colonization and mortality rates [26].

In the present study we were also assessing the dynamics of inflammatory responses in the course of infection with two different *A*. *butzleri* strains. Whereas the *A*. *butzleri* reference strain CCUG 30485 had been initially isolated from a diseased patient [21], the C1 strain was derived from fresh chicken meat [8]. It is therefore not known if the latter is also able to induce disease in humans. Overall, the most distinct influx of immune cell populations such as T cells, Tregs, monocytes and macrophages into the colonic lamina propria could be observed six days following CCUG 30485 strain infection which was paralleled by peaking concentrations of pro-inflammatory mediators such as TNF, IFN-γ, NO and IL-6 in the large intestines, and IFN-γ and IL-12p70 in serum samples of infected mice declining therafter until day 16 p.i. Interestingly, other pro-inflammatory cytokines such as TNF and IL-6 increased later in the course of infection with either strain and were higher at day 16 as compared to day six p.i., pointing towards differentially regulated immune responses upon *A*. *butzleri* infection. It is hence highly likely that different *A*. *butzleri* strains might exert distinct host-dependent immune responses given that some strains cause overt disease in humans whereas others act as commensals like in chicken, for instance [31]. Furthermore, *in vitro* assays revealed differences in adhesive and invasive potentials of several *A*. *butzleri* strains [8, 9, 32]. However, no correlation between these phenotypes and the corresponding virulence gene pattern or functional domains of adhesion and invasion associated genes could be determined [8, 9]. Nevertheless, the *A*. *butzleri* strains CCUG 30485 and C1 applied in our gnotobiotic IL-10^-/-^ model, displayed a similar pattern of virulence genes and comparable adhesive and invasive capabilities in *in vitro* assays [8]. Taken together, eventhough both strains stably colonized the murine large intestines they induced slightly distinct local and systemic host responses in infected gnotobiotic IL-10^-/-^ mice. This suggest that further, so far unraveled, virulence associated genes might be encoded by *A*. *butzleri*, contributing to different immunopathological potencies of respective *A*. *butzleri* strains.

In conclusion, *A*. *butzleri* induces not only intestinal but also extra-intestinal and systemic immune responses in gnotobiotic IL-10^-/-^ mice following peroral infection. This points towards an immunopathogenic and differentially regulated role of *A*. *butzleri* in vertebrate hosts. Moreover, these results highlight gnotobiotic IL-10^-/-^ mice as a valuable infection model to further unravel the underlying molecular mechanisms of *A*. *butzleri* induced pathogenicity in the near future.

## Supporting Information

S1 Table Raw data for fecal shedding of *A*. *butzleri* strains in orally infected gnotobiotic IL-10^-/-^ mice. (XLSX)

S2 Table Raw data for apoptotic and proliferating cells in colon of gnotobiotic IL-10^-/-^ mice following *A*. *butzleri* colonization. (XLSX)

S3 Table Raw data for colonic immune cell response following *A*. *butzleri* infection of gnotobiotic IL-10^-/-^ mice. (XLSX)

S4 Table Raw data for colonic pro-inflammatory mediators responses following *A*. *butzleri* infection of gnotobiotic IL-10^-/-^ mice. (XLSX)

S5 Table Raw data for nitric oxide secretion in renal *ex vivo* biopsies of *A*. *butzleri* infection of gnotobiotic IL-10^-/-^ mice. (XLSX)

S6 Table Raw data for systemic pro-inflammatory cytokine responses in *A*. *butzleri* infection of gnotobiotic IL-10^-/-^ mice. (XLSX)
